# Pilot Study: PARP1 Imaging in Advanced Prostate Cancer

**DOI:** 10.1007/s11307-022-01746-w

**Published:** 2022-06-14

**Authors:** Farrokh Dehdashti, Melissa A. Reimers, Kooresh I. Shoghi, Delphine L. Chen, Jingqin Luo, Buck Rogers, Russell K. Pachynski, Sreeja Sreekumar, Cody Weimholt, Dong Zhou

**Affiliations:** 1 Edward Mallinckrodt Institute of Radiology, Washington University School of Medicine, 510 South Kingshighway Blvd, St. Louis, MO 63110 USA; 2 Department of Internal Medicine, Alvin J. Siteman Cancer Center, Washington University School of Medicine, St. Louis, MO USA; 3 Department of Radiology, University of Washington, Seattle, USA; 4 Seattle Cancer Care Alliance, Seattle, USA; 5 Department of Surgery, Alvin J. Siteman Cancer Center, Washington University School of Medicine, St. Louis, MO USA; 6 Department of Radiation Oncology, Washington University School of Medicine, St. Louis, MO USA; 7 Department of Pathology and Immunology, Alvin J. Siteman Cancer Center, Washington University School of Medicine, St. Louis, MO USA

**Keywords:** PET, Positron emission tomography, PARP, Cancer, PARP inhibitor (PARPi) therapy, FTT

## Abstract

**Purpose:**

PARP inhibitor (PARPi) therapy is approved for patients with metastatic castration-resistant prostate cancer (mCRPC) and homologous recombination repair (HRR) genomic aberrations. However, only a fraction of patients with *BRCA1/2* mutations respond to PARPi therapy. In this pilot study, we assess PARP-1 expression in prostate cancer patients with and without HRR genomic alternations using a novel PARP-based imaging agent.

**Procedures:**

Nine advanced prostate cancer patients were studied with PET/CT and [^18^F]FluorThanatrace (FTT), an analogue of the PARPi rucaparib. Images were analyzed using maximum standardized uptake values (SUV_max_). PARP expression was assessed by immunohistochemistry (IHC) when feasible (*n* = 4).

**Results:**

We found great variability in FTT uptake (SUV_max_ range: 2.3–15.4). Patients with HRR mutations had a significantly higher SUV_max_ (*p* = 0.0379) than patients with non-HRR mutations although there was an overlap in FTT uptake between groups. Three patients without HRR and one with HRR mutations had similarly high PARP1 IHC expression.

**Conclusions:**

FTT-PET/CT may serve as an alternate biomarker for PARP1 expression and a potential method for PARPi treatment selection.

## Introduction

Prostate cancer (PC) is one of the most common malignancies in men. However, despite multiple new therapeutic options, the overall survival for metastatic prostate cancer (mPC) has only improved by about 7 months over the last decade [1]. DNA repair pathways, including homologous recombination repair (HRR), are critical to maintaining cellular integrity. Double-strand DNA breaks can be repaired via either homologous recombination or non-homologous end-joining (NHEJ) [2]. Multiple genes, importantly *BRCA1* and *BRCA2*, play a key role in HRR. Furthermore, poly(ADP-ribose) polymerase 1 (PARP-1) is required for single-strand DNA break repair [3, 4]. PARP-1 binds to DNA at sites of DNA breaks and recruits poly(ADP-ribose) (PAR) to facilitate DNA repair [5]. PARP inhibitors (PARPi) impair this process of DNA repair via synthetic lethality and other mechanisms such as PARP trapping on DNA, leading to cell death [6, 7]. However, the frequency of HRR genomic aberrations in PC is relatively low, and potentially actionable somatic HRR mutations are present only in about 20% of mPC patients [8–10]. Nonetheless, a randomized clinical trial demonstrated improved progression-free and overall survival with the PARPi olaparib versus those who received androgen receptor targeted therapy in mCRPC patients with *BRCA1/2* or *ATM* mutations [11, 12]. Thus, novel biomarkers beyond HRR mutations are needed to identify additional PC patients who may respond to PARPi.

Several PET radiotracers to image PARP expression have recently been developed [13]. [^18^F]FluorThanatrace (FTT), and ^18^F-PARPi bind to PARP1, are derived from rucaparib and olaparib, respectively [13], and are currently being evaluated in breast and ovarian cancers [14, 15]. FTT has been developed at our institution, and we have previously reported that FTT has an optimal biodistribution in humans, and the primary route of excretion is through hepatobiliary system [16]. The normal organs with the highest uptake were the pancreas, spleen, and liver. The objective of the current pilot study was to evaluate the dynamic range of PARP1 expression in prostate cancer patients with and without HRR genomic alternations utilizing FTT-PET.

## Materials and Methods

### Study Design and Participants

This study was part of a prospective, single-site phase 0 clinical trial to demonstrate the feasibility of imaging PARP1 expression with FTT-PET/CT in cancer patients (NCT02469129, IND 124,116). The study was approved by all institutional regulatory committees and performed according to the Declaration of Helsinki and the rules of Good Clinical Practice guidelines. All subjects gave written informed consent for participation. Trial eligibility for this investigational imaging study was broad; patients with any type of cancer planned to undergo surgical resection/biopsy, or who were planned to receive systemic therapy, were eligible. Subjects were required to have measurable disease per RECIST 1.1 on CT or other standard radiological imaging. Measurable disease was defined as one or more tumor sites measuring > 1 cm in the shortest transaxial diameter. Subjects with disease < 1 cm were ineligible. Patients who had claustrophobia or who were unable to lie in the PET scanner were also excluded. Vital signs and laboratory evaluations (CBC, CMP) were performed before and after completion of FTT-PET/CT for assessment of any clinically significant change that may have been related to FTT administration. Subjects also were closely monitored during the study for any symptoms that may have been related to FTT injection.

**Fig. 1:**
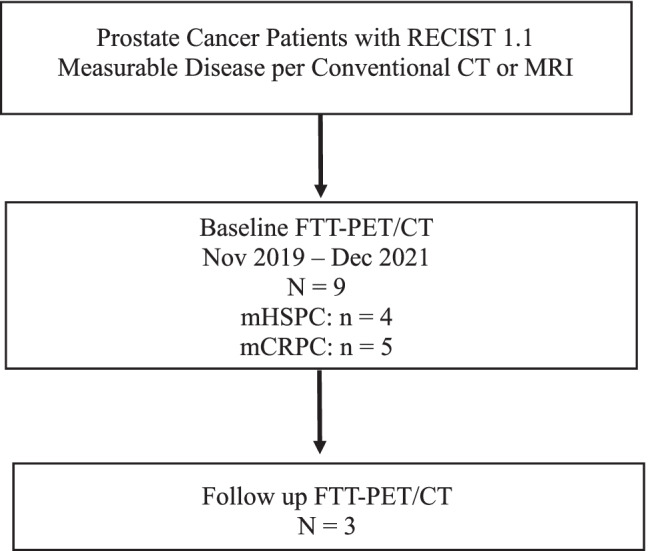
Study schema. mHSPC = metastatic hormone sensitive prostate cancer, mCRPC = metastatic castration resistant prostate cancer.

For the present study, patients were consented under the NCT02469129 protocol and were recruited from advanced PC patients in our clinical practice who were either treatment naïve or were scheduled to start a new treatment regimen (Fig. 1). Four patients had metastatic hormone-sensitive prostate cancer (mHSPC); two patients had de novo metastatic disease, and two had recurrent metastatic disease. The remaining 5 patients had metastatic castration-resistant disease (mCRPC) (Table 1). All patients underwent standard of care bone scintigraphy and computed tomography (CT) at baseline prior to enrollment for measurable disease assessment prior study enrollment. All patients also underwent [^18^F]fluorodeoxyglucose (FDG)-PET/CT) before or after FTT-PET/CT (mean interval 3.6 days). Subjects were encouraged to undergo follow-up imaging after therapy (minimum of 3 months after therapy), but in our cohort, only three patients (#3, 6 and 8) underwent FTT-PET/CT after therapy (Table 1).

**Table 1:** Patients characteristics

Patient # (age)		PSA (ng/mL) at FTT-PET	Tumor histology (GS)	Therapy prior to imaging	Therapy following Imaging	PARP1 IHC scores	Genomic aberrations and biopsy site
“WT” mutations	1 (56)	355.10	*de novo mHSPC (5 + 4 = 9)	Treatment naïve	ADT and docetaxel	8	*RB1* LOF (left acetabulum)
	2 (79)	30.47	de novo mHSPC (4 + 5 = 9)	Treatment naïve	ADT and docetaxel	8	*TP53* LOF (liver)
	4 (63)	12.89	mCRPC 4 + 3 = 7	No therapy (on break from ADT)	ADT and apalutamide	N/A	Heterozygous *RAD50* pathogenic mutation (peripheral blood^)
	6 (71)	73.10	mCRPC (4 + 4 = 8)	ADT	Olaparib and ADT	8	*CDK12* LOF (parasternal soft tissue mass),*ATM* (0.2% by ctDNA¥, peripheral blood)
“MUT” mutations	3 (76)	88.86	mCRPC (4 + 5 = 9)	ADT	Olaparib and ADT	7	Somatic *BRCA2* copy number loss (iliac bone)
	5 (80)	7.85	mCRPC (5 + 4 = 9)	ADT	Docetaxel and ADT	N/A	*ATM* LOF (lymph node)
	7 (66)	17.46	mCRPC (4 + 3 = 7)	Olaparib	Cabazitaxel	N/A	Heterozygous germline *BRCA2* pathogenic variant (peripheral blood#)
	8 (67)	44.72	mHSPC (4 + 3 = 7)	pTVG-HP and pembrolizumab	Olaparib and ADT	N/A	Germline *BRCA2* pathogenic variant and somatic *BRCA2* copy number loss (LOH) (sacrum)
	9 (62)	130.10	mHSPC (4 + 4 = 8)	Docetaxel and ADT	Olaparib and ADT	N/A	Germline *BRCA2* pathogenic variant (adrenal)

*MUT BRCA1/2* and *ATM* pathogenic variants, *WT* all other mutations, *GS* Gleason score, *mHSPC* metastatic hormone sensitive prostate cancer, * de novo mHSPC with neuroendocrine features, *mCRPC* metastatic castration-resistant prostate cancer, *ADT* androgen deprivation therapy, *LOF* loss of function, *LOH* loss of heterozygosity, *ctDNA* circulating tumor DNA prostate cancer. ^Germline analysis via Ambry Genetics assay. #Germline analysis via Myriad Genetics. ¥ctDNA analysis via Guardant 360; pTVG-HP = pTVG-HP is a plasmid DNA, produced in *E. coli*, that encodes the complementary deoxyribonucleic acid (cDNA) for human prostatic acid phosphatase (PAP). Administered as part of a clinical trial with pembrolizumab

### Genomic Analysis

Baseline metastatic biopsy was not required for study entry, and 7 of 9 patients had metastatic tissue available for genomic analysis. Standard of care next-generation sequencing (NGS) was performed utilizing the Tempus xT 648 platform with matched normal blood analysis for germline testing (Tempus Labs; Chicago, IL, USA, https://www.tempus.com; Table 1). Two of the 9 patients did not have metastatic tissue available and thus underwent peripheral blood analysis for germline genetic analysis with the Ambry and Myriad platforms, respectively. One patient also underwent additional ctDNA testing with Guardant 360 in addition to tissue NGS at a separate timepoint prior to receiving care at our institution (Table 1). Four of the 9 patients had sufficient tissue for PARP1 expression analysis via immunohistochemistry (IHC). For this study, we classified *BRCA1/2* and *ATM* mutations, the genomic aberrations most associated with response to PARPi, as “MUT,” and all other mutations as “WT”.

### Imaging and Analysis

FTT was prepared as previously described in our cyclotron GMP facility using the GE FXNPro module under current good manufacturing practice (cGMP) guidelines [14]. Briefly, FTT was synthesized under cGMP conditions using a GE FXN Pro reaction module via nucleophilic substitution of a tosylate precursor with a solution of pre-dried [^18^F]KF/K2CO3/Kryptofix2.2.2 in DMSO at 105 °C. The crude FTT was then purified via a reversed phase HPLC (Zorbax SB-C18, 250 × 9.4 mm, mobile phase 15% acetonitrile (0.1% TFA) in water). The isolated peak was diluted in 40 mL of sterile water for injection (SWI), trapped on an HLB cartridge, eluted from the cartridge using 0.9 mL of ethanol (200 Proof), and formulated in normal saline to provide a 10% ethanol/saline solution. This was sterile filtered through a 0.2-micron Millex-GV filter directly into a sterile final product vial. The final product was analyzed by HPLC, GC, pH, filter membrane integrity test, appearance, color, radiochemical identity, bacterial endotoxin test, and sterility was confirmed as a post release test. All release criteria were met for all injections [14, 16]. The final product meets the releasing criteria approved by FDA.

Patients underwent a 60-min dynamic study immediately following intravenous administration of FTT (median 370 MBq, range 336.7–388.5) over the largest and/or the previously biopsied lesion. Emission imaging were then obtained extending from the skull base to the upper thighs. FTT-PET images were evaluated for areas of abnormal tracer uptake in comparison with anatomical, bone scintigraphy and FDG-PET/CT images using tumor maximum standardized uptake value (SUV_max_). For each of the 9 patients, the SUVs of 5 lesions (typically, the largest) were determined, and a total of 45 different disease sites were evaluated.

### Immunohistochemistry (IHC) Staining

Biopsy samples were analyzed for PARP1 using an immunohistochemistry (IHC) staining protocol previously described by our group with modifications [17]. Briefly, formalin-fixed paraffin-embedded tissue Sects. (5 µm) were deparaffinized, rehydrated, and subjected to antigen retrieval (citrate buffer, pH 6.0). The sections were blocked (10% normal goat serum), and immunostained with PARP1 (46D11) rabbit antibody (1:300; #9532, Cell Signaling Technologies, USA) followed by ImmPRESS goat anti-rabbit secondary antibody (Vector Laboratories, USA). Diaminobenzidine (Agilent Dako, USA) was used as a chromogen, and sections were counterstained with hematoxylin, dehydrated, cleared, and mounted with Cytoseal XYL (Thermo Scientific, USA). Images were captured using a BX51 microscope (Olympus, Japan). Scoring was performed by an experienced genitourinary pathologist blinded to the clinicopathologic data of the patients. Scoring was adapted from the Allred model of breast cancer estrogen receptors/progesterone receptor staining, whereby an IHC score is calculated as the sum of the positive proportion (0 = 0% positive tumor cells; 1 =  < 1%; 2 = 1–10%; 3 = 11–33%; 4 = 34–66%; 5 = 67–100%) and the staining intensity (0 = no staining; 1 = weak; 2 = moderate; 3 = strong) for a possible total score of 8. PARP1 was considered positive if the IHC score was greater than 4.

### Statistical Analysis

Demographic and clinical characteristics were summarized by descriptive statistics. The linear mixed effects model for repeated measures was applied, separately to FTT SUV_max_ in raw and log scales, to model the effect of gene mutation while accounting for the repeated measures of tumor SUV_max_ for the same patient. The model residuals were examined for goodness of fit, which showed better model fitting using the SUV_max_ in log scale. The least square mean for MUT and WT was reported with associated standard error and Wald test *P* value testing the estimated mean against 0. The least square mean difference between MUT (*n* = 5) and WT (*n* = 4*)* patients was reported with associated standard error, the Wald test *p* value testing the mean difference against 0, and the type III test *p* value comparing the model with mutation versus the null model without.

## Results

Nine PC patients were enrolled (median age of 67 years, range 56–80). Patient characteristics are summarized in Table 1. From a radiotracer safety perspective, no clinically detectable pharmacological effects of FTT or changes in the results of laboratory studies were observed—that is, patients in this study did not have any appreciable alterations in blood chemistries or vital signs after FTT administration.

**Fig. 2:**
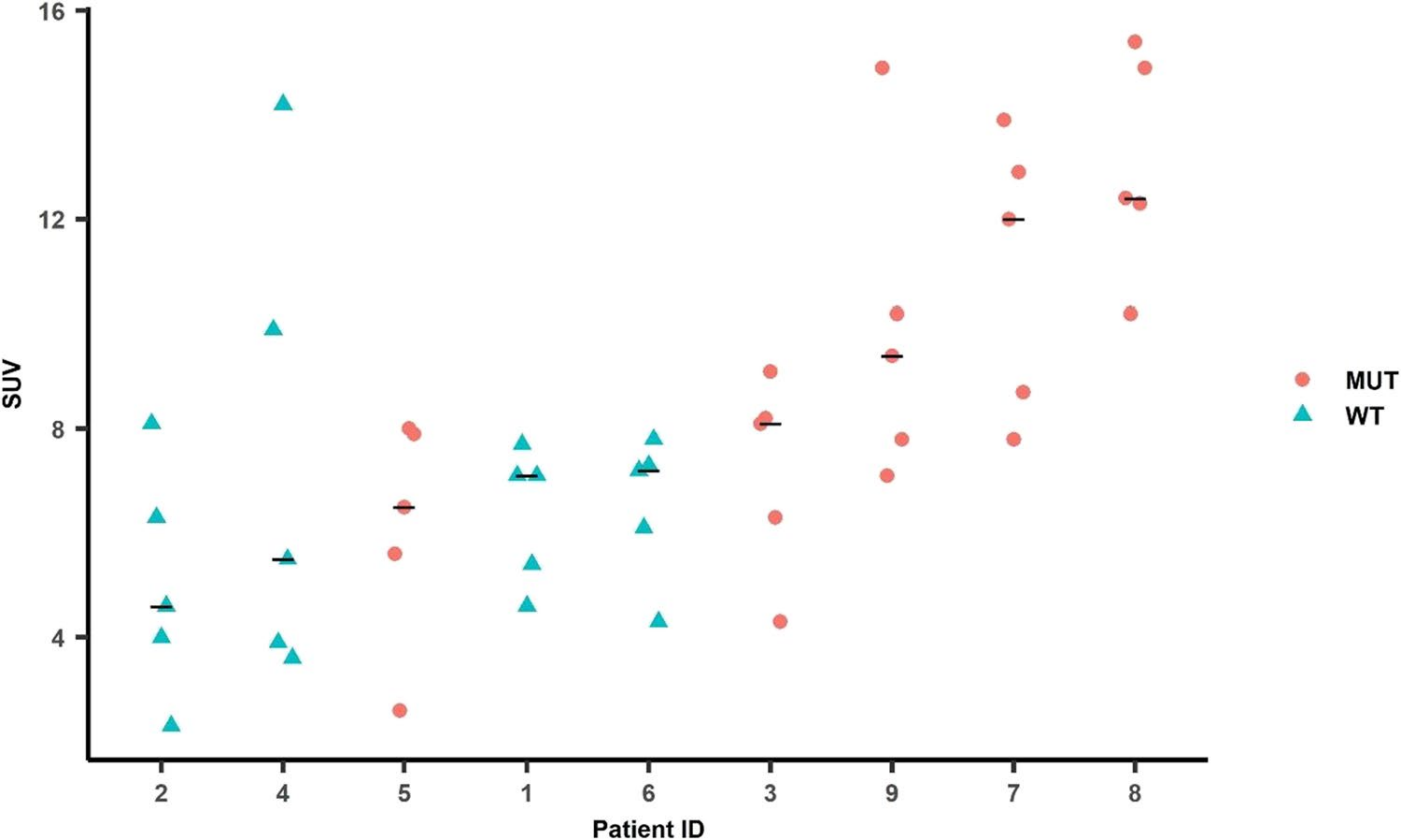
The graph is patient ID on the x-axis plotted against FTT SUV_max_ on the y-axis. Patients are arranged on the x-axis in ascending order of FTT SUV_max_. Red dots indicate MUT tumor lesions and cyan triangles indicate WT tumor lesions. The black horizontal line indicates the median SUV_max_ across all lesions for a given patient. There was a statistically significant difference between the SUV_max_ of the MUT (*n* = 5) and WT (*n* = 4) patients (*p* = 0.0379). MUT, *BRCA1/2*, *ATM*; WT, non-*BRCA1/2* or *ATM* mutations.

The imaging data were derived from 45 different disease sites: bone (*n* = 18), liver (*n* = 2), prostate/prostate bed (*n* = 6), adrenal gland (*n* = 1), and lymph nodes (*n* = 18) (Figs. 1 and 2). As would be expected for advanced prostate cancer, a broad range of organs were involved with metastatic disease: 2 patients had osseous and lymph node metastases, one had osseous and soft tissue, one had osseous and hepatic, 1 had osseous and adrenal gland, 1 had osseous, and 1 had lymph node metastases. The SUV uptake for each of the studied lesions is delineated in Table 2.

**Table 2:** Summary of semiquantitative analysis (maximum SUV) in individual patients

Patient	FDG/FTT (Lesion Site #1)	FDG/FTT (Lesion #2)	FDG/FTT (Lesion #3)	FDG/FTT (Lesion #4)	FDG/FTT (Lesion #5)
1	4.3/7.1 (Acetabulum)	4.3/7.7 (Pelvic LN)	1.8/4.6 (Retroperitoneal LN)	3.1/5.4 (L4)	5.1/7.1 (Prostate)
2	8.3/8.1 (Prostate)	12.9/6.3 (Hepatic)	10.9/4 (iliac)	6.4/4.6 (Ischium)	4.3/2.3 (Pubis)
3 (*BRCA2*)	13.6/8.1 (Femur)	5.2/8.2 (Ilium)	6.3/9.1 (L5)	4.0/6.3 (Sacrum)	1.9/4.3 (Supraclavicular LN)
4	3.5/9.9 (Paracaval LN)	4.3/3.9 (Common Iliac LN)	2.8/14.2 (Pre-Rectal LN)	3.9/5.5 (Internal Iliac LN)	3.5/3.6 (Para-aortic LN)
5 (*ÂTM*)	6.3/5.6 Inguinal LN)	43.2/2.6 (Prostate)	9.9/6.5 (Obturator LN)	3.2/8.0 (Para-aortic LN)	2.8/7.9 (Supraclavicular LN)
6 (*ATM, CDK12*)	9.1/7.2 (Sternal/Para-sternal soft tissue)	2.6/6.1 (Prostate)	1.2/7.3 External Iliac LN)	1.4/7.8 (Prevascular)	0.7/4.2 (Obturator LN)
7 (*BRCA2*)	8/13.9 (Perirectal LN)	21.1/12 (Retroperitoneal LN)	9.4/7.8 (Parasternal)	27.8/12.9 (Pericardial LN)	8.6/8.7 (Hepatic)
8 (*BRCA2*)	16.6/12.4 Prostate	15.1/10.2 L5	2.4/15.4 Humerus	4/12.3 Ischium	16.3/14.9 Spinous process
9 (*BRCA2*)	9.8/14.9 Adrenal	5.9/7.1 Sternum	8/9.4 T3	13.4/10.2 Humerus	6.8/7.8 Sacrum

*LN* lymph node

**Fig. 3:**
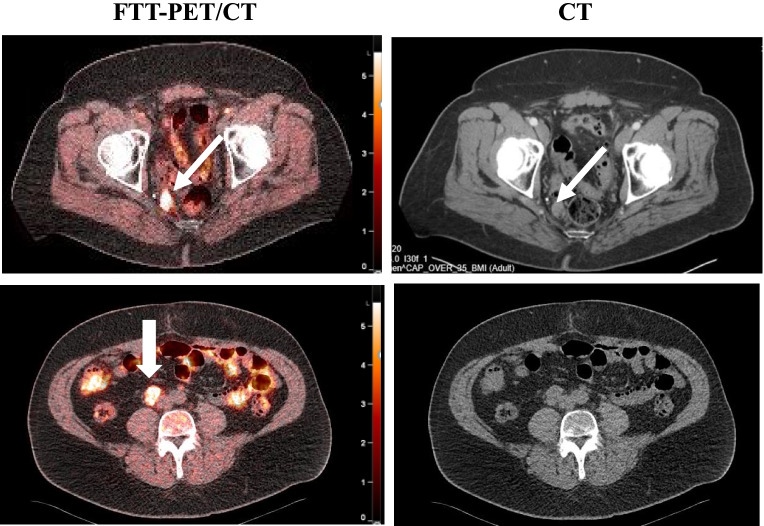
Prostate adenocarcinoma (#4). Fused FTT-PET/CT images (left) and CT images (right). There are FTT + right perirectal (top row) and right internal iliac lymph nodes (bottom row arrows). The scale bar on PET images: SUV_max_ = 5.5

**Fig. 4:**
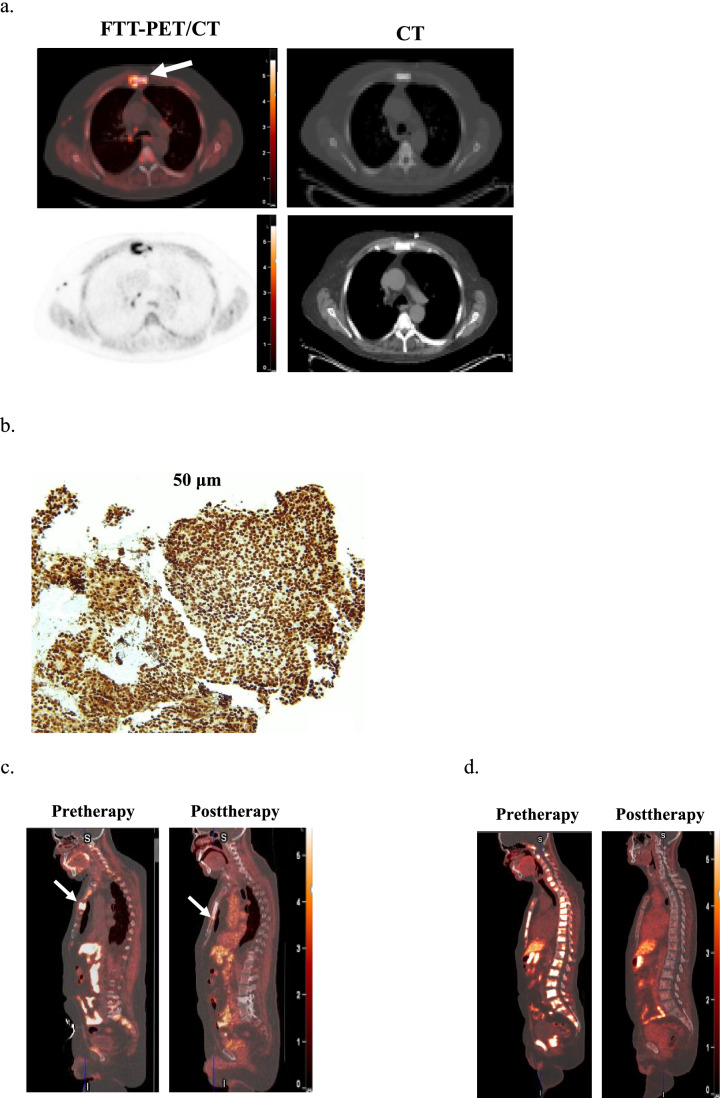
Prostate adenocarcinoma (#6). **A** Fused FTT-PET/CT (left top row) and CT (right: top, bone window; bottom, soft tissue window) images. There is FTT + sternal metastasis (arrows) with slightly different distribution. Additional FTT + mediastinal lymph nodes are noted. **B** PARP1 IHC score of 8. **C** Fused FTT-CT sagittal images of before and 4 months after PARPi therapy (#6 non-responder). The post-therapy image demonstrates the resolution of the sternal lesion (arrow) seen on pre-therapy image and appearance of a new sternal lesion just inferior to the initial lesion (arrow). **D** Fused FTT-CT sagittal images of before and 4 months after PARPi therapy (#8 responder). The post-therapy image demonstrates near-complete resolution of all spine and sternal metastatic disease. The scale bar on PET images: SUV_max_ = 5.5

The SUV_max_ for FTT ranged from 2.3 (similar to normal bone) to 15.4 in osseous lesions (Fig. 2). We did not identify a significant difference in SUV_max_ between osseous (mean and range 8.82; 3.6–14.2) and lymph nodes/soft tissue lesions (7.50; 2.3–15.4, *p* = 0.6471). After linear mixed effects modeling of FTT SUV_max_ on a log scale (log scale FTT SUV_max_ difference mean/SE = 0.4306/0.1685), patients with MUT genomic alterations had a significantly higher (Wald test *P* = 0.0379) FTT SUV_max_ than patients with WT alterations (Fig. 2). Patients 3, 9, 7, and 8, all of whom harbored either germline or somatic *BRCA2* alterations, had the highest FTT uptake. Although patient 4 (Fig. 3), who harbored an *ATM* LOF mutation and was categorized as MUT, the uptake for this patient was proportionally lower compared with the *BRCA* mutant patients. The patients with the lowest SUV_max_ harbored *TP53*, *RB1*, *RAD50*, and *CDK12* mutations. Of note, both patients 3 (*BRCA2* copy number loss) and patient 8 (simultaneous *BRCA2* germline alteration and somatic mutation) underwent a baseline FTT PET and then initiated treatment with standard of care olaparib. Follow-up FTT-PET/CT was completed at 9 and 10 months (Fig. 4), respectively. At the time of the follow-up imaging, the PSA markedly decreased in patient 3 (88.86 to 0.90 ng/mL) and patient 8 (44.72 to 0.90 ng/mL), correlating with excellent radiographic response.

Patient 6 had only one site of known disease (sternum) on CT, FDG-PET/CT and bone scintigraphy and several additional sites of FTT avid osseous and lymph node disease, which were suspected to be additional sites of disease. He was initiated on olaparib therapy due to a *CDK12* tumor mutation and an *ATM* mutation on cell-free DNA liquid biopsy. However, the *ATM* variant allele fraction (VAF) was very low at 0.2%, suggesting a likely subclonal alteration. On post-therapy scan 5 months later, the patient’s PSA increased (4.61 ng/mL to 8.26), correlating with disease progression on FTT-PET/CT and correlative imaging. The mean SUV of these lesions were lower than that in patients # 3 and # 8 (6.5 vs. 7.2 and 13.04, respectively) who responded to PARPi therapy (Fig. 4).

IHC analysis demonstrated high PARP1 expression (score 5 + 3 = 8 in 3 WT patients and 5 + 2 = 7 in 1 MT patient), correlating with high FTT uptake (Table 1, Fig. 4).

## Discussion

Despite six FDA-approved treatments for mCRPC, long-term survival outcome in advanced PCa remains poor, and further treatment advances are needed. mCRPC with HRR genomic aberrations have been shown to be sensitive to PARPi [11, 18]. However, based on the PROfound trial, patients with non-*BRCA/ATM* genomic aberrations (*BRIP1*, *BARD1*, *CDK12*, *CHEK1*, *CHEK2*, *FANCL*, *PALB2*, *PPP2R2A*, *RAD51B*, *RAD51C*, *RAD51D*, or *RAD54L*) do not benefit to the same degree as patients with alterations in *BRCA1/2* or *ATM* (response rate to olaparib: 4% vs. 33%, respectively) [19, 20]. These mixed results have also been demonstrated in the TRITON2 trial, wherein patients with deleterious *BRCA1/2* alterations had greater response to rucaparib than patients with *ATM*, *CHEK2*, and *CDK12* mutations [18, 21]. Thus, the role of HRR genomic aberrations in accurately predicting response to PARPi remains unclear. Therefore, it is of critical importance to identify a means for selecting patients likely to respond to PARPi that is not solely dependent on evaluating HRR status. In our study, patients harboring either somatic or germline *BRCA2* genomic alterations had the highest FTT uptake, and two of these patients also demonstrated radiographic response on follow-up FTT-PET that correlated with clinical PSA response to PARPi therapy. However, particularly noteworthy is that fact that the patient with a CDK12 mutation who received olaparib did not have a favorable response on FTT-PET. This lack of clinical benefit correlates with the lack of radiographic benefit reported in TRITON2 in *CDK12* patients (0 of 10 patients), suggesting that lack of high FTT uptake may be predictive of low likelihood of response to PARPi [22].

Currently, there are several ongoing clinical trials of FTT-PET/CT in breast, ovarian, pancreatic, brain, and prostate cancers. Several studies have shown the specificity of FTT for in vivo quantitative imaging of PARP1 [15, 23]. Similar results have been reported in breast cancer patients where FTT uptake was independent of breast cancer subtypes *BRCA* pathogenic variant carriers and non-carriers [24]. Our study demonstrates the feasibility of FTT-PET/CT in PC patients as a potential imaging biomarker to augment patient selection appropriate for PARPi, and we found a wide range of FTT uptake independent of HRR mutation status.

Furthermore, one patient with MUT and 3 patients with WT had PARP1 IHC analysis and demonstrated high expression, suggesting that tumor suppressor gene loss may also be associated with PARP1 expression. Alternatively, this may simply be confirming highly variable PARP-1 IHC regardless of BRCA status in patients with ovarian, breast, and prostate cancers, as shown by others [25–27]. Thus, an *in viv*o imaging biomarker such as FTT uptake may serve as a putative biomarker for potential response to PARPi and may be a preferred mode in selection of appropriate patients for PARPi therapy. The main limitation of our study is the small sample size. Additional subjects are needed to fully characterize the utility of the FTT as an imaging biomarker for selection of patients who will respond to PARPi therapy. In addition, the correlation between PARP1 expression and response to therapy independent of established HRR genomic aberrations need to be evaluated.

## Conclusion

In the present pilot study, we demonstrated the feasibility of imaging advanced PC patients with FTT-PET/CT. The studied lesions had a broad range of SUV uptake independent of HRR status, but with overall higher uptake in patients harboring BRCA2 mutations. Our preliminary results support further investigation of FTT-PET/CT as a technology to evaluate PARP1 expression in vivo, with potential application as a biomarker for PARPi treatment selection in advanced PC.
